# Characterizing provider-led adaptations to mobile phone delivery of the Adolescent Transition Package (ATP) in Kenya using the Framework for Reporting Adaptations and Modifications to Evidence-based Implementation Strategies (FRAME-IS): a mixed methods approach

**DOI:** 10.1186/s43058-023-00446-y

**Published:** 2023-08-14

**Authors:** Dorothy Imbuka Mangale, Alvin Onyango, Cyrus Mugo, Caren Mburu, Nok Chhun, Dalton Wamalwa, Irene Njuguna, Arianna Rubin Means, Grace John-Stewart, Bryan J. Weiner, Kristin Beima-Sofie

**Affiliations:** 1 Department of Global Health, University of Washington, Seattle, USA; 2 Research & Programs, Kenyatta National Hospital, Nairobi, Kenya; 3 Department of Epidemiology, University of Washington, Seattle, USA; 4 Department of Paediatrics and Child Health, University of Nairobi, Nairobi, Kenya; 5 Department of Pediatrics, School of Medicine, Seattle, USA; 6 Department of Medicine, University of Washington, Seattle, USA; 7 Department of Health Services, University of Washington, Seattle, USA

**Keywords:** Adaptation, Implementation study, Implementation strategy, COVID-19, mHealth, Adolescents, Youth, FRAME-IS

## Abstract

**Background:**

The COVID-19 pandemic resulted in disruptions to routine HIV services for youth living with HIV (YLH), provoking rapid adaptation to mitigate interruptions in care. The Adolescent Transition to Adult Care for HIV-infected adolescents (ATTACH) study (NCT03574129) was a hybrid I cluster randomized trial testing the effectiveness of a healthcare worker (HCW)-delivered disclosure and transition intervention — the Adolescent Transition Package (ATP). During the pandemic, HCWs leveraged phone delivery of the ATP and were supported to make adaptations. We characterized real-time, provider-driven adaptations made to support phone delivery of the ATP.

**Methods:**

We conducted continuous quality improvement (CQI) meetings with HCWs involved in phone delivery of the ATP at 10 intervention sites. CQI meetings used plan-do-study-act (PDSA) cycles and were audio-recorded. Adaptations were coded by two-independent coders using the Framework for Reporting Adaptations and Modifications to Evidence-based Implementation Strategies (FRAME-IS). Adaptation testing outcomes (adopt, retest, or abandon) and provider experience implementing the adaptations were also recorded. We summarized adaptation characteristics, provider experience, and outcomes.

**Results:**

We identified 72 adaptations, 32 were unique. Overall, adaptations included modification to context (53%, *n* = 38), content (49%, *n* = 35), and evaluation processes (13%, *n* = 9). Context adaptations primarily featured changes to personnel, format, and setting, while content and evaluation adaptations were frequently achieved by simple additions, repetition, and tailoring/refining of the phone delivery strategy. Nine adaptations involved abandoning, then returning to phone delivery. HCWs sought to increase reach, improve fidelity, and intervention fit within their context. Most adaptations (96%, *n* = 69) were perceived to increase the feasibility of phone delivery when compared to before the changes were introduced, and HCWs felt 83% (*n* = 60) of adaptations made phone delivery easier. Most adaptations were either incorporated into routine workflows (47%) or tested again (47%).

**Conclusion:**

Adaptation of phone delivery was a feasible and effective way of addressing challenges with continuity of care for YLH during the COVID-19 pandemic. Adaptations were primarily context adaptions. While FRAME-IS was apt for characterizing adaptations, more use cases are needed to explore the range of its utility.

**Trial registration:**

Trial registered on ClinicalTrial.gov as NCT03574129.

**Supplementary Information:**

The online version contains supplementary material available at 10.1186/s43058-023-00446-y.

Contributions to the literature This paper illustrates how team-based learning sessions in the form of CQI meetings can be utilized by frontline healthcare workers to systematically develop and apply adaptations to quickly adjust to changing context (i.e., COVID-19). This is useful for researchers exploring methods for integrating dynamic adaptation into routine healthcare worker workflow in constrained settings.

This paper demonstrates the retrospective application of FRAME-IS to characterize adaptations, highlighting an approach to further our understanding of implementation through the study of adaptation types and their characteristics.

This paper adds to the literature on the use of qualitative methods for studying adaptations and underscores the potential for framework or rapid analysis of qualitative data to quickly identify and characterize adaptations or adaptation traits associated with improved implementation outcomes in real time.

## Background

The COVID-19 pandemic presented one of the most challenging health emergencies in recent history [1]. Disruptions were widespread, and movement restrictions posed unique challenges to health facilities around the world, particularly in low- and middle-income countries (LMICs) [2]. Vulnerable populations, including adolescents and young adults (AYA), were especially at risk of prolonged service disruptions [3]. AYA are also disproportionately affected by the HIV epidemic, making up 30% of incident HIV globally [4]. Interferences to HIV care due to COVID-19 posed a threat to HIV outcomes within this age group, especially older AYA aged 19–24 years who demonstrate worse outcomes than their younger counterparts [5, 6]. Strategies were urgently needed to avoid or mitigate service disruption to HIV care for this group. The use of digital technology to support provision of remote or virtual HIV services via mHealth (including telemedicine or telehealth) emerged as a common and important strategy for addressing pandemic-related service disruptions [7–12]. This offered a unique opportunity to examine the implementation of mHealth and its determinants when introduced to safeguard access to health services during emergencies.

The specific benefits of mHealth for adolescent HIV services are well known but how these interventions are implemented is less studied [13–16]. In the pandemic, the deployment of mHealth solutions was rapid, sometimes without guidelines, prior assessments of suitability to the context, or adequate formal training of healthcare workers (HCWs) to guide implementation. This kind of rapid implementation necessitated iteration of products or services to fit provider and client needs, as well as the context. Adaptations are formally defined as planned and purposeful changes made to the interventions or delivery strategies while maintaining the integrity of the core components or processes associated with the relevant effectiveness outcomes [17, 18]. Adaptations differ from modifications which are typically viewed as unplanned or spontaneous changes [19]. Although adaptations are intended to introduce improvements, when forced or introduced hurriedly, they may compromise service delivery and effectiveness by lowering feasibility and fidelity of implementation [20]. Studies have shown that HCWs are sometimes not systematic in the development of planned adaptations [21–24]. This is common where cultural adaptations are required to meet diverse needs but evidence on core components of the intervention or strategy is unknown [24]. Many adaptations generated during the pandemic, especially those targeting healthcare workers were ad hoc, making replication and comparison of adaptation procedures across implementation units challenging [7, 10, 25, 26]. Furthermore, the rapid nature of adaptation development may render the adaptations difficult to identify or test and may have a negative impact on implementation fidelity.

The importance of methodically detailing and understanding adaptations has been underscored in recent literature [27–29]. More recently standardized frameworks and language describing ways to develop, document, and describe adaptations have become available [30–34]. These tools offer ways to group and study adaptations by important elements such as the motivation, level of rationale, change idea involved, and targeting of core versus peripheral components of interventions or strategies. Lack of, or partial characterization and comprehension of adaptations limits our ability to identify and prioritize sustainable changes, and impedes the advancement of evidence on how mechanisms of change associated with specific services or interventions lead to outcomes.

The Adolescent Transition to Adult Care for HIV-infected adolescents (ATTACH) study was a cluster randomized trial (NCT03574129) that tested the effectiveness of an Adolescent Transition Package (ATP) in 20 HIV clinics in Kenya [35]. The ATP aimed to prepare youth with HIV as they transition to independent care by increasing HIV knowledge and self-management skills required for independent care [36]. In this trial, frontline HCWs applied continuous quality improvement (CQI) meetings and plan-do-study-act (PDSA) cycles to develop dynamic, provider-led adaptations to phone delivery of the ATP following the onset of the COVID-19 pandemic. In this paper, we characterize the adaptations using the expanded Framework for Reporting Adaptations and Modifications for Implementation Strategies (FRAME-IS) [34]. FRAME-IS provides a consistent approach for describing adaptations. FRAME-IS is derived from FRAME, which specifically focuses on categorizing adaptations to interventions, and can be applied to guide the adaptation process [31]. It comprises four core and three optional modules useful for describing adaptations. The modules document (1) a description of the evidence-based intervention, the implementation strategy, and adaptation; (2) a description of what was modified to obtain the adaptation; (3) the nature of the modification and whether it maintains fidelity to the core components; (4) the goal of the adaptation and the level of the system that had most sway in selection of the adaptation; (5) when in the phases of implementation the adaptation occurred, and if it was planned; (6) specification of individuals or groups participating in the decision to adapt; and (7) the spread of the adaptation within the context of implementation [34]. This paper tests the applicability of FRAME-IS for describing context-specific adaptations made during a pragmatic trial and demonstrates how the framework’s classification system may support the identification of patterns in adaptations selected. We also summarize feedback on implementing the adaptations after a brief testing period. Describing adaptations to the use of digital technology may highlight changes that can inform future actions, in analogous scenarios.

## Methods

### Setting and study design

This sub-study combines qualitative and quantitative data from the ATTACH study, which was a hybrid cluster randomized controlled trial (RCT) conducted in 4 counties in Kenya (Nairobi, Homa Bay, Kajiado, and Nakuru) [35]. Participants were recruited at 20 HIV comprehensive care clinics (CCCs) selected based on clinic location, size (> 500 clients in care, and ≥ 50 YLH in care), use of electronic medical records, and approval of facility leadership. CCCs are sites for the delivery of coordinated HIV care for adult, adolescent, and pediatric populations. Adolescent HIV care at participating sites is provided free of charge and the CCCs were typically staffed by clinical officers, medical officers, nurses, adherence counselors, and link officers, among others [37]. Ten clinics were randomized to receive the intervention, the ATP, while ten maintained the standard of care.

The ATP includes HCW tools to support the transition process, including a booklet (Taking Charge) designed to guide patient education and empowerment sessions during YLH clinic visits and a tracking tool to document YLH progress through the booklet. The intervention was designed to be delivered in-person by CCC HCWs. HCWs were trained to walk through the booklet step by step to facilitate YLH learning. After each exposure to the booklet, progress was noted in the tracking tool. The trial tested the effectiveness of the ATP and found that those receiving the intervention had significantly higher overall transition readiness scores and higher scores in HIV literacy domain compared to those in the control arm [36]. When COVID-19 social distancing and movement restrictions were put in place, the study pivoted from in-person to phone delivery of the ATP. HCWs made phone calls to YLH once a month for up to one hour to deliver the contents of the ATP. Calls were to be made during work hours at a time determined by each individual HCW. During calls, HCW introduced themselves and checked if YLH were ready to talk. HCW then read through and discussed relevant sections of the ATP.

This study was a partnership between the University of Washington, Kenyatta National Hospital (KNH), and University of Nairobi (UoN). Research ethics approval was granted by the University of Washington Institutional Review Board (UW IRB) and the Kenyatta National Hospital/University of Nairobi Ethics and Review Committee (KNH/UoN ERC). The study protocol is available online (https://pubmed.ncbi.nlm.nih.gov/33268417/). Findings in this paper were reported in accordance with Standards for Reporting Implementation Studies checklist [38].

### Participants

HCWs were recruited from among clinic staff who work with YLH at all intervention sites. To enhance the representativeness of the study, we purposively sampled individuals from various cadres including clinical officers, nurses, physicians, counselors, and psychologists. All HCWs involved with the care of YLH at each selected to participate. Willing HCWs provided written consent, emphasizing voluntary participation and the option of opting out at any point without repercussions. HCWs joining clinic sites or clinic activities after the trial began were informed about the study and provided written consent.

### Data collection

We collected qualitative data to identify and characterize adaptations and quantitative data to describe the CQI meeetings and participation. We facilitated CQI meetings using PDSA cycles to optimize ATP implementation by phone at ten intervention sites between June 29^th^ and 19^th^ of October 2020. Twice monthly CQI meetings were conducted over 4–5 months with each site completing at least 5 CQI cycles by the end of the adaptation period. A final CQI meeting was held to review all adaptations and gather HCW perspectives on the feasibility, ease, and success of implementing the adaptations. CQI meetings were led by study staff. During meetings, CCC HCWs identified changes to phone delivery of the ATP, evaluated previous adaptations (if any), reflected upon adaptation effectiveness, and decided to adopt, retest, or discontinue an adaptation (adaptation outcomes). The meetings were audio-recorded, and study staff completed PDSA worksheets during meetings to document key elements of the discussion.

We also collected quantitative data to describe the implementation of the phone strategy. For each call made to an adolescent, the following data points were recorded in a call log: when the call was made, who the call was made to, what time the call was made, in what environment the call was received, what chapter of the ATP was discussed and whether the call was successful.

### Data processing

Adaptation data were extracted from audio files by a primary analyst and entered in a REDCap-based form which mirrored modules of FRAME-IS and FRAME because adaptations identified in this study were related to both the intervention and the implementation strategy. The select modules were (1) what is being modified, (2) the nature of the modification, (3) the main goal and rationale for the modification, and (4) the spread of the adaptation. Where audibility was a challenge, we utilized CQI facilitator notes and CQI recordings from each site’s final CQI meeting during which all changes made by each site underwent a final review. PDSA worksheets were also consulted in a few circumstances where audio recordings were of poor quality. Most meetings concluded with the identification of one adaptation but some sites selected multiple adaptations per cycle. Two independent primary coders (DM and NC) reviewed each adaptation, classifying adaption characteristics according to framework domains. Together, analysts reviewed the agreement in their coding and discussed convergence and divergence in their understanding of the adaptations. We also categorized adaptations based on whether they focus on the implementation strategy (phone delivery), the evidence-based intervention (the ATP), or both.

### Data analysis

We used descriptive statistics to summarize CQI occurrence and duration, call characteristics, adaptation features, and adaptation outcomes using medians and ranges for continuous variables, and sums and proportions for binary or categorical variables. To analyze the adaptation data, we categorized the qualitative data and quantified it. We reported the number of adaptations to phone ATP delivery reported overall and by site, the feature or aspect targeted for adaptation, adaptation types, reasons for adaptations, level of rationale for the adaptation, and the groups targeted by the adaptation. We summarized the adaptation characteristics and the proportion of adaptations focused solely on the implementation strategy (phone delivery), the evidence-based intervention (the ATP), or both.

## Results

Over a 4-month period following the introduction of phones for delivery of ATP, 59 CQI meetings were held to facilitate the adaptation process (average of 6 per facility) (Table 1). Meetings included a median of 5 (range: 4–10) frontline HCWs per site and involved a variety of cadres (nurses, clinical officers, peer educators, mentor mothers/fathers, adherence and HIV testing counselors, nutritionists, link officers, and records officers) primarily from CCC and prevention of mother to child transmission clinics. CQI meetings took a median of 21 min (range 13–75).

**Table 1:** Summary of CQI meetings and adoption of the ATTACH phone call strategy

CQI summary	
Post-COVID CQI meetings	59
HCW per CQI (median)	5 [4-10]
Duration (median, minutes)	21 [13–75]
Phone call adoption success	
Calls attempts	*N* = 1444
Calls reaching AYA	82% (1180)
Calls successful	96% (1137)
Phone charged	99% (1175)
Call received in a conducive environment	96% (1148)
Day call was made	*N* = 566
Weekday	88% (498)
Weekend/public holiday	12% (68)
Time call was made	*N* = 566
Morning	28% (158)
Afternoon	57% (323)
Evening	15% (85)
At least one chapter discussed	*N* = 1180
	93% (1096)

HCWs made 1444 call attempts, 82% (*n* = 1180) of which reached YLH. Ninety-six percent (*n* = 1137) of attempted calls were successful, meaning the HCW was able to discuss relevant ATP content with the adolescent. Most calls were made on weekdays (88%, *n* = 498), in the afternoon or evening (82%, *n* = 408), and nearly all calls, 93%, covered at least one chapter of the ATP tool.

### Adaptation characteristics

We identified 72 adaptations, 32 of which were unique and mainly targeting the actor or implementer of the phone strategy to improve on HCW organization, workflow, and patient-HCW engagement. Tables 2 and 3 summarize unique adaptations and their characteristics. Most of the adaptations were simple and grounded in one main idea, however, there were a number that comprised multiple ideas but communicated as one adaptation.

**Table 2:** Summary of phone strategy adaptations identified across sites during CQI meetings

	Adaptations	Frequency
Action	Wait for the next scheduled clinic visit/Default to in-person	5
	Provide shortened time to the next in-person clinic visit	1
	Schedule calls in advance, increase airtime, and shuffle HCWs	2
	Simplify language	1
	Add interactive elements	1
	Obtain/use alternate contact information	7
	Define notation for documenting client progress	1
	Total	16
Actor	Divide adolescents among HCWs	10
	Designate a specific cadre responsible for follow-up calls	1
	Reduce staff making calls and create targets	1
	Match HCW to client based on client language needs and assess understanding	4
	Use CHV/link person/community drug distribution to locate adolescent	7
	Create priority list to organize HCW access to phone	1
	Prioritizing HCWs going on leave and handing over pending clients	1
	Shuffling HCWs who are making calls	2
	Assign a specific cadre to oversee proper documentation	1
	Total	28
Setting	Calling after hours	3
	Carry phone home for afterhours calls	1
	Total	4
Target	Ask clients to document important details	1
	Target calls to caregivers	3
	Assign clients memorization activities and check recall	2
	Create a priority list based on adolescents with the fewest call attempts and schedule	1
	Implementing pre-calls to schedule and prepare adolescents	8
	Total	15
Timing	Call at different times of day	2
	Postponing calls during heavy workload seasons	1
	Designating call days	1
	Total	4
Frequency	Repetition of ATP chapters	2
	Repeating call attempts	4
	Increase volume of calls attempts made per day	1
	Total	7
Dose	Spreading material over several calls	1
	Reduce material covered during call and match clients’ language needs	1
	Total	2

Frequencies represent unique or first-time appearances of an adaptation at each site. If a facility sustains a unique adaptation, it is not counted repeatedly. Some adaptations are a combination of > 1 change and may target different components of the phone strategy

**Table 3:** Summary of the application of FRAME-IS AND FRAME to the ATTACH Phone Strategy

The EBP being implemented is: The Adolescent Transition Package	
The Implementation Strategy being modified is: the phone delivery strategy	
Specification of the implementation strategy	
Action: Making a phone call	
Actor: Frontline HCW at CCC clinics	
Context/Setting: CCC clinics	
Target: YLH	
Timing: During working hours (8am–5 pm)	
Frequency: Once a month	
Dose: One chapter per call for up to 45 min	
The modifications being made are:	See Table 1 and Appendix 1
The reasons for modification are:	Low phone ownership
	Incorrect phone numbers
	Challenges incorporating calls to workflow
	Low-quality call experience
	Fear of unintended disclosure
	Scheduling conflict
	(See Appendix 1 for extensive list)
What is modified:	Context (format, personnel, and setting)
	Content
	Evaluation procedures
What is the nature of the content, evaluation, or training modification?	Adding, tailoring, spreading
Relationship to core elements:	NA
What is the goal:	Increase adoption
	Increase reach
	Increase fit
	Increase feasibility
	Increase fidelity and effectiveness
What is the level of the adaptation rationale?	Implementer and patient level
When is the modification initiated?	Mid-implementation phase
Is the modification planned?	Planned/proactive
Who participants in the adaptation decision?	Entire team of implementers/care team
How widespread is the modification?	All implementers and patients
	Sub-sets of implementers and patients who share similar characteristics

**Fig. 1:**
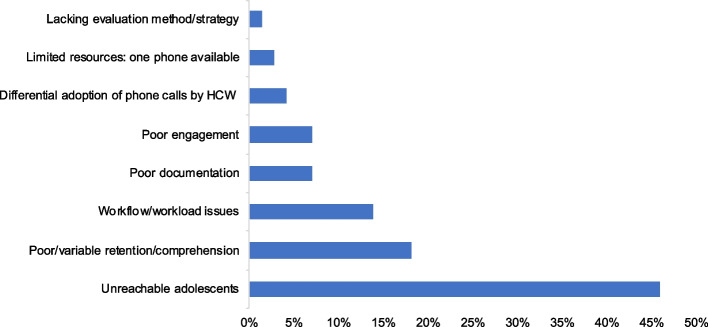
Reasons for adaptations across all intervention sites (*n* = 10)

The reasons for adaptations were varied and constituted challenges in implementing the phone strategy or reaching YLH. HCWs most frequently adapted how they were implementing phone delivery to address barriers such as difficulties reaching YLH because of missing or incorrect contact information, scheduling challenges, low phone ownership in this group, limited availability of youth, and fear of unintended disclosure which manifested in YLH refusing or ending calls without warning (Fig. 1, Appendix 1). The phone strategy had limited success where YLH were sharing phones with unsupportive caregivers, siblings, or partners. HCW-level barriers that drove the decision to adapt were also identified. For example, initially, some HCWs did not consider making phone calls part of their job. Only one phone was available to HCWs at each site, forcing HCWs to share access. HCWs also experienced burdensome workloads, especially during reporting periods when the majority of their time was spent collating files, documenting, and enumerating outcomes. Decline in staffing numbers due to COVID-19 infection was also common.

**Fig. 2:**
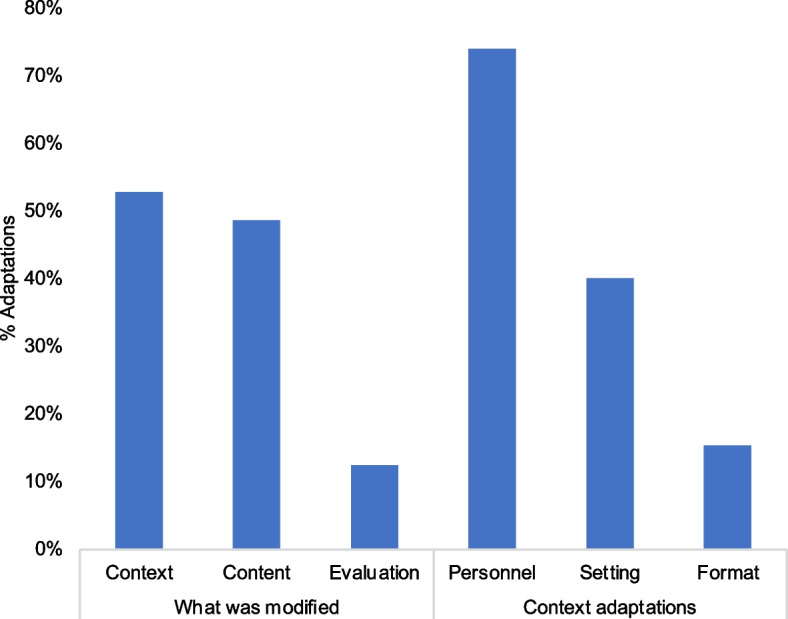
Targets of adaptations across all intervention sites (*n* = 10)

Adaptations were primarily context adaptions (53%, *n* = 38); 28 adaptations involved only context changes (Fig. 2, Appendix 1) These featured personnel (74%, *n* = 26) and setting (40%, *n* = 14) changes affecting the overall way the phone strategy was delivered. Personnel-related context adaptations included sharing of tasks among available staff, prioritizing phone use for HCWs based on an agreed-upon schedule, or redistribution of tasks to HCWs with more availability, or with better rapport or language skills compatible with specific YLH.

**Fig. 3:**
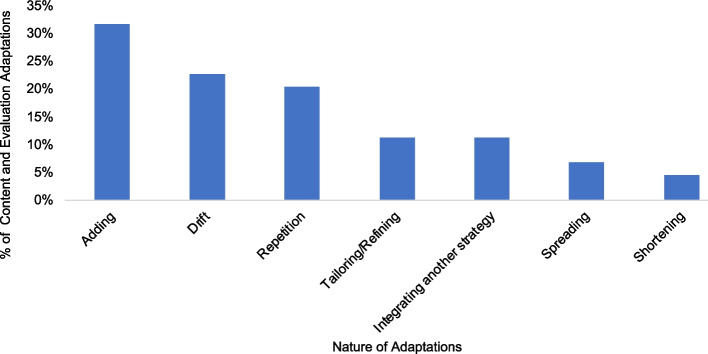
Nature of content and evaluation adaptations across all intervention sites (*n* = 10)

The frequency of content adaptations was similar to context adaptations (49%, *n* = 35, with 28 exclusively classified as such). Lastly, we noted 13% (*n* = 9) evaluation adaptations to respond to the need to document and evaluate the progress YLH made during calls. The nature of content and evaluation adaptations ranged from addition of discrete elements like questions or the use of scripts (32%, *n* = 20), focused refinements or tailoring (8%, *n* = 5), repetition for example repeating calls or repeating call content (15%, *n* = 9), shortening (2%, *n* = 1), spreading material over several calls (5%, *n* = 3) and integration of home tracing using community health volunteers as another strategy alongside the phone strategy (8%, *n* = 5; Fig. 3). There were several instances of drift with return (8%, *n* = 5) to the original strategy. For example, when YLH could not be reached at all HCWs resorted to inviting them to attend clinic visits to discuss the ATP.

**Fig. 4:**
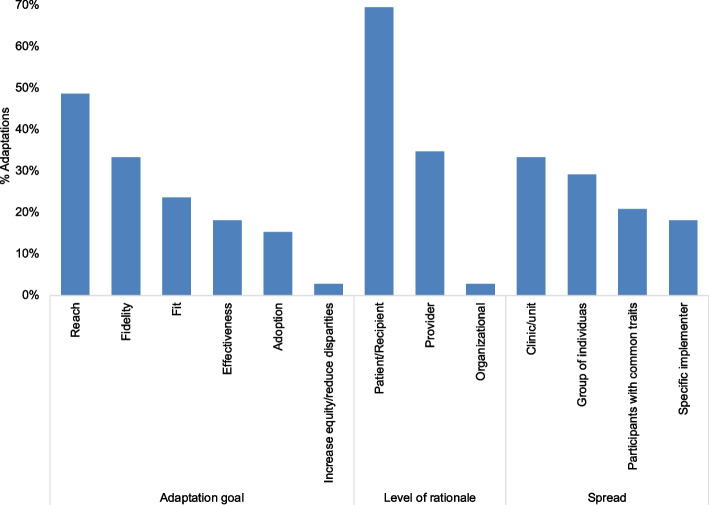
Goals, level of rationale, and spread of adaptations across all intervention sites (*n* = 10)

The goals of the adaptations were most frequently to increase reach (49%, *n* = 35), fidelity of implementation (33%, *n* = 24), and fit of the strategy to the HCWs’ schedules, preferences, environment, and workload (24%, *n* = 17; Fig. 4). Improving effectiveness of the intervention was less frequently discussed and, in some instances, co-occurred with the fidelity goal. The same was noted with adoption, which co-occurred with a few discussions about improving the reach and fit of the strategy.

The level of the organization that most directly informed the adaptations was the recipient level (69%, *n* = 50), aligning with the goal of increasing the number of calls reaching YLH (Fig. 4). There was also much focus on adaptation rationale from the HCW level (35%, *n* = 25) which influenced the selection of modifications that aimed at improving adoption by removing or reducing workflow-related barriers. We noted that majority of adaptations were intended to reach all HCWs in the implementing unit (33%, *n* = 24) and all YLH clients receiving the ATP intervention (29%, *n* = 21; Fig. 4). Targeted adaptations were present but less frequent: 21% (*n* = 15) for youth with specific characteristics or issues and 18% (*n* = 13) for individual implementers whose specific role, skills, or experience were leveraged to address client- or HCW-level barriers.

### Site-level differences

**Fig. 5:**
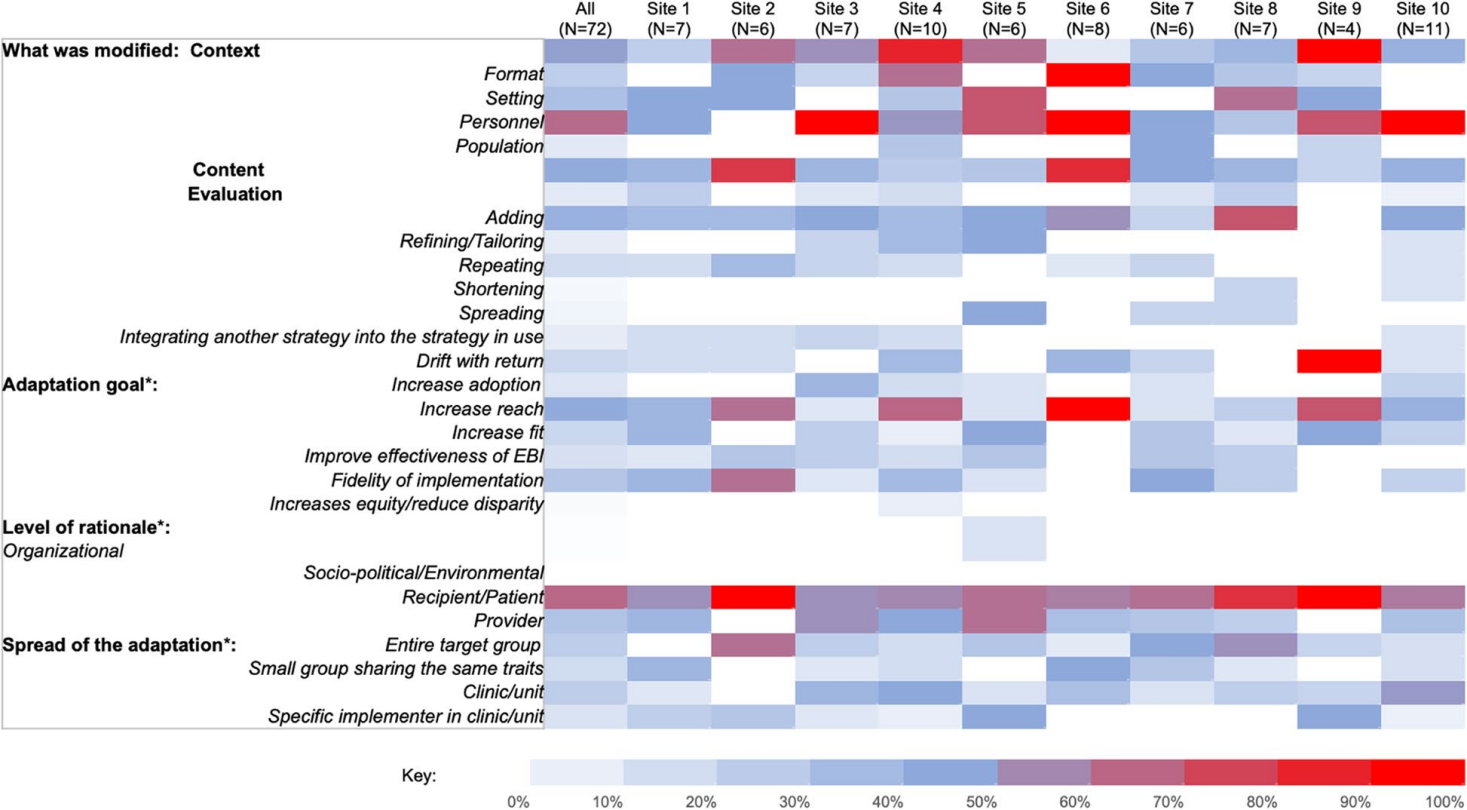
Heatmap summarizing characteristics of adaptations, overall and by site

Comparison of adaptations across sites demonstrated that sites were tackling similar challenges to phone-based ATP delivery and conceived of similar adaptations. A few differences were in the number of adaptations per site: the median number of adaptations per site was 7 (range: 4–11) and some variation across sites in what was modified. For example, one facility implemented context adaptations exclusively while another had 25% context modifications and 75% content modifications. For all facilities, most context modifications involved format changes except for one facility that primarily implemented personnel-related adaptations. Only six facilities enacted evaluation adaptations. Figure 5 summarizes the variation in adaptation characteristics across sites.

### Outcomes of adaptation implementation

HCWs perceived that 96% (*n* = 69) of adaptations made implementation of the phone strategy more feasible than before the change was introduced. Overall, 83% (*n* = 60) of the adaptations were perceived to make delivery of the phone strategy easier than when there was no adaptation (Table 4). Temporary or permanent migration of adolescents, phones that were turned off, wrong numbers and a reduction in number of staff available to make calls due to COVID-19 infection are examples of factors that made phone delivery of the ATP less successful. After testing, most adaptations were either incorporated immediately into routine workflows (47%) or selected for further testing and then adopted after secondary review (47%) (Table 4). Very few adaptations were abandoned after testing (6%): for example, home visits that leveraged community health volunteers (CHVs) were abandoned at one site because of lack of funds to support that cadre’s activities.

**Table 4:** Outcomes of implementing adaptations to the phone strategy, overall and by site

*N* = number of adaptations	All (*N* = 72)	Site 1 (*N* = 7)	Site 2 (*N* = 6)	Site 3 (*N* = 7)	Site 4 (*N* = 10)	Site 5 (*N* = 6)	Site 6 (*N* = 8)	Site 7 (*N* = 6)	Site 8 (*N* = 7)	Site 9 (*N* = 4)	Site 10 (*N* = 11)
*Implementation of adaption went as planned:*	96% (69)	86% (6)	100% (6)	100% (7)	100% (10)	100% (6)	100% (8)	100% (6)	100% (7)	75% (3)	91% (10)
*Phone delivery after adaptation easier than in-person:*	83% (60)	86% (6)	100% (6)	71% (5)	100% (10)	100% (6)	63% (5)	83% (5)	71% (5)	75% (3)	91% (10)
*Decisions made after adaptation testing:*											
* Adapt*	47% (34)	14% (1)	67% (4)	86% (6)	40% (4)	50% (3)	25% (2)	50% (3)	71% (5)	25% (1)	27% (3)
* Adopt*	47% (34)	86% (6)	33% (2)	14% (1)	60% (6)	50% (3)	38% (3)	50% (3)	14% (1)	75% (3)	73% (8)
* Abandon*	6% (4)	-	-	-	-	-	38% (3)	-	14% (1)	-	-

HCWs recommended the use of the WhatsApp platform to augment the phone strategy by facilitating individual or group communication, sharing of ATP text and pictures, and allowing storage of information in text form so that adolescents can refer to it when needed. Other recommendations from HCWs included providing shorter times to clinic appointments, providing phones to clients, increasing airtime allocation, and increasing the number of phones available to HCWs. The use of video calls was suggested to improve the remote visit experience. HCWs also felt increasing call frequency may help adolescents retain what they have learned.

## Discussion

The objective of our study was to characterize dynamic, provider-led adaptations to phone delivery of the ATP using FRAME-IS. We found that adaptation was a feasible way of addressing barriers to continuity of transition support care for YLH during the early stages of the COVID-19 pandemic. FRAME -IS was a useful tool for characterizing and understanding adaptations (summarized in Table 3). Adaptations were similar across all sites and originated from 32 unique ideas. The inability to reach clients was a recurrent reason for adaptations. The need for adaptations emerged from challenges integrating the phone delivery strategy into their workload and workflow. We identified primarily context adaptations which were frequently intended for the whole care team or all YLH receiving the intervention. To our knowledge, this is the first study in our setting to characterize adaptations using FRAME-IS.

In our study, the main motivation for proposing adaptations was to improve the reach of the phone strategy. HCWs were unable to reach adolescents for a variety of reasons. For example, when called, YLH were busy, with many competing interests which resulted in scheduling conflicts. There were also frequent refusals to answer calls or abrupt ending of calls because of fear of unintended disclosure. Limited phone ownership was also a common challenge in this population and many YLH were reliant on phones owned by a caregiver, sibling, or partner. Uptake of the phone strategy was impacted by scheduling conflicts with caregivers or the unwillingness of caregivers, sibling, or partner to support the client by availing the phone at necessary times, for a sufficient duration, or fostering privacy and confidentiality needed by the adolescents. These and other barriers appear elsewhere in literature examining the implementation of digital technologies for health [39, 40]. Additionally, the role of stigma on youth engagement with health services is not new and has an impact on adoption and uptake of health services in the clinic setting as well as virtually [41, 42]. Our study demonstrates how using CQIs facilitated the identification of challenges and the selection of adaptations to address them successfully.

Addressing barriers in the HCW workflow was another important motivation for adaptations to phone delivery. At each facility, HCW were sharing one phone to implement the strategy and were not always able to complete their call loads when scheduled at the same time as other colleagues. The addition of new activities to their workflow at times conflicted with pre-existing responsibilities. For instance, HCWs found it hard to adopt the phone strategy during monthly reporting periods when the majority of their time was required to collate reports. Key adaptations useful for improving reach beyond the trial were scheduling calls ahead of time and incorporating separate preparatory calls targeted to caregivers. HCW identified these changes as priorities for long-term integration into their routine activities. This trial exemplified how frontline HCWs in our context can be engaged in adaptation selection and testing to mitigate challenges in accessing health services. This was especially important during the pandemic when health systems were facing staffing shortages due to illness or redistribution of HCWs to areas of highest need, interruptions in the supply chain, and reallocation of financial resources.

Most adaptations involved context changes, that is tweaking the way the phone strategy was delivered. In implementation science, context refers to the constellation of cultural, organizational, social, financial, and leadership aspects that influence how an innovation or strategy is provided or received. Given that all these spheres were affected by the pandemic in one way or another, the frequency of context adaptations is not unexpected [25, 43, 44]. For example, task shifting aspects of phone delivery to community health workers was a context adaptation to address gaps in reaching adolescents when they failed to answer calls or lacked contact information in their patient files.

The rest of the adaptations we identified were primarily content changes achieved by making simple additions or refinements to the intervention. In our scenario, these kinds of simple adaptations may represent opportunities for optimization that are low-lying-fruit as they neither required extensive time nor resources and would introduce minimal disruption to the overall functioning of the health system. Simple adaptations make sense where HCWs are not trained in the language and concepts of adaptations or improvement science. Nonetheless, it was highly advantageous to involve frontline HCWs, including members of informal cadres like mentor mothers or fathers, in the adaptation process. Their proximity to clients, opportunity for rapport-building, and familiarity with clients provided access to information necessary to customize and optimize phone delivery of the ATP to their needs and contexts. Engaging community perspectives is important for leveraging local knowledge and expertise to create programs that are relevant and customized to the target population, to encourage trust-building, and to promote positive and sustainable involvement in facility-based care [19, 45, 46].

Post-CQI evaluations underscore the acceptability, feasibility, and appropriateness of the adaptations as most of them were either adopted immediately after testing or adapted and then adopted after a second round of testing. However, the occurrence of drifts from the strategy cannot be ignored: we identified nine instances where the adaptation involved abandoning the phone strategy because previous adaptations were infeasible or unsuccessful. For example, the use of CHVs was limited by a lack of funds to support their commute. The phone strategy was also abandoned when HCW identified certain individuals’ inability to retain information, or who were virally unsuppressed and required further clinical examination. The limited capacity of telemedicine for physical examination, HCW-patient relationship-building, and as a medium for patient education has been noted as a persistent barrier and fuel for HCWs’ resistance to the use of this and other digital health strategies [40, 43, 47].

Of note, the use of WhatsApp to deliver the ATP intervention was proposed up to three times across all CQI meetings but not selected for testing in the PDSA cycle. HCWs in non-clinical cadres who are primarily tasked with retaining clients in care noted that communication via WhatsApp had been effective for maintaining contact, information sharing including appointment reminders, and engaging youth in support groups. The feasibility and success of using WhatsApp for reaching adolescents has been identified in several studies [41, 44, 48]. The novelty of the application, affordability of data required to operate it, capacity for text and voice messages, video calls, and sharing of documents, exemplify the youth-friendliness of the platform, making it a promising avenue for improving the ATTACH phone strategy and other mHealth interventions. More work is needed to explore the best ways of utilizing this technology to support YLH transitioning out of pediatric care.

FRAME-IS is the first tool developed specifically to address modifications and adaptations to implementation strategies. Its modular structure and flexibility make it an apt and systematic tool for classifying the adaptations. Few studies have applied FRAME-IS to track and/or characterize adaptations [49–54], but more examples are needed to explore the range of its utility for Implementation Science and to identify areas of improvement. Currently, FRAME-IS is not designed to capture or describe adaptation outcomes and may benefit from incorporating a module that does this. An outcomes module may inspire a more careful selection of adaptations. Within LMICs, tracking outcomes may provide the information needed by frontline HCWs in LMICs to focus adaptations. One study merged FRAME-IS and RE-AIM frameworks so that HCWs could leverage reach and adoption outcomes to track progress following a given adaptation [50]. This allowed the teams to compare between adaptations and select for the high-yield ones. Future work may focus on cataloging adaptations that support the best yield in clinics, including their FRAME-IS-defined characteristics. This resource would introduce efficiencies to adaptation development by allowing HCWs to focus on identifying solutions that fit their context or reconfiguring adaptation characteristics of select ideas to improve fit and feasibility in their clinics. Incorporating more instructions and a user-friendly design to guide the process of adaptation selection and specification may increase use of FRAME-IS among adaptation teams. This could support real-time documentation and characterization of adaptations and may offer more benefits to implementers than the retrospective application of the framework. Furthermore, there is room to strengthen our understanding of adaptations and their downstream influence on outcomes by adding another module that documents micro-contextual changes coinciding with the introduction of adaptations, for example when trained HCWs transfer out or a champion for the strategy emerges within the care team.

This study used PDSA protocols to structure the adaptation process, thereby introducing a degree of methodological rigor. Guided by research staff who introduced in simple terms the process and importance of this approach, HCWs engaged in consensus discussions to identify context-specific adaptations. However, we observed that leading and coaching teams to identify specific change concepts or ideas was challenging. The implementation team may have benefited from broad knowledge about the range of changes possible and linkages between barriers and adaptations that are most suited to address them.

Although we were able to characterize the adaptations, we noted that the coding process was challenging. Coding directly from audio-recordings to structured forms defining FRAME-IS modules was not a 1:1 process; it involved condensing a lot of information to fit the discrete modules. In some cases, overlap in the categorization of adaptations was unavoidable. The groups within the modules did not always allow for parsimonious characterization such that some adaptations fell into several categories at the same time. Additionally, it was challenging to apply it to adaptations that were an amalgamation of several change ideas e.g., an adaptation that consists of an additional element delivered at a different frequency by a different cadre of HCW. This may impact our ability to recognize patterns or associations in adaptation data when examining and comparing across similar studies. Lastly, the categorization of adaptations was retrospective, and coding was done by researchers. Real-time, prospective characterization of the adaptations using forms or checklists modeled after FRAME-IS may be an advantageous approach, especially in the context of a pandemic or other emergencies. We note that rapid qualitative analysis using FRAME-IS as a coding guide may be a game-changer in ensuring that adaptation data can be fed back to facilities and policy makers within a shorter timeline [55, 56].

### Study limitations

The extraction and characterization of adaptation data happened retrospectively and relied primarily on audio recordings that were of variable quality. However, we overcame this issue by looking to data in PDSA forms for clarification. CQI process differed from site to site. There was a degree of subjectivity in the way the meetings were structured and run, which depended on the ATTACH staff. For example, not all teams discussed what to do with the adaptation after two weeks of testing during the meeting. In some cases, the decision was made and captured two or three CQI meetings later. It is possible that the experiences and variation of adaptations presented in this study may not mirror what is possible in other contexts. In this study we attempted to track implementation outcomes linked to each adaptation using a survey, however, due to pandemic-related protocol changes these data were incomplete and insufficient for inclusion. We also did not specify core versus peripheral elements of the phone strategy to HCWs nor assess their fidelity to the implementation strategy. Therefore, we were unable to ascribe changes in study outcomes to specific adaptations.

## Conclusion

Knowledge that interventions are not always effective when transferred to new contexts provides impetus for further study of the role of adaptations. The need for rapid adaptation is also underscored in significant emergencies like the COVID-19 pandemic which introduce sudden changes in the immediate context. To study adaptations, it is important to identify, document, and evaluate them, while considering the dynamism of implementation contexts. In this study, we saw how the introduction of adaptations allowed frontline HCWs to introduce and implement solutions that impacted their ability to adopt the phone delivery strategy. We identified and characterized a range of challenges associated with phone delivery of the ATP, which may occur at other facilities if we were to scale out to new clinics. Using FRAME-IS we classified what were a majority of context adaptations to address these gaps. This study adds to the literature describing a range of possible adaptations which allowed HCWs to maintain engagement with clients during the COVID-19 pandemic when many health systems experienced prolonged service interruptions. Additionally, it demonstrates the utility of FRAME-IS and adds to existing examples of its application in research.

### Supplementary Information

**Additional file 1: Appendix 1. **Adaptations Coding

## Data Availability

The processed data supporting the conclusions of this article are included within the article (and its additional file(s)). The source data from the study are available from the corresponding author on reasonable request.

## Abbreviations

YLH: Youth living with HIV
ATP: Adolescent Transition Package
ATTACH: Adolescent Transition to Adult Care for HIV-infected adolescents
HCW: Healthcare worker
CQI: Continuous quality improvement
PDSA: Plan Do Study Act
FRAME-IS: Framework for Reporting Adaptations and Modifications to Evidence-based Implementation Strategies
CCC: Comprehensive care clinic
